# Long-term changes of metal contents in two metallophyte species (Olkusz area of Zn-Pb ores, Poland)

**DOI:** 10.1007/s10661-016-5330-3

**Published:** 2016-05-10

**Authors:** Alicja Kicińska, Agnieszka Gruszecka-Kosowska

**Affiliations:** Faculty of Geology, Geophysics and Environmental Protection, Department of Environmental Protection, AGH University of Science and Technology, Mickiewicza 30 av., 30-059 Kraków, Poland

**Keywords:** Zn-Pb smelters, *Agrostis capillaris*, *Betula pendula*, Heavy metals, Metallophytes

## Abstract

The authors present the changes of the As, Cd, Fe, Mn, Pb and Zn contents in two plant-considered metallophytes: common bent *Agrostis capillaris* (blades) and birch *Betula pendula* (leaves and seeds), recorded in a Zn-Pb industrial region of Olkusz (Poland) in 1994 and 2014. The highest amounts of Cd (12 ppm) and Zn (2524 ppm) in the common bent occur in the vicinity of the mining and metallurgical works ‘Bolesław’ in Bukowno. However, these values are significantly lower than those in 1994: Cd by 87 % and Zn by 52 %. The highest contents of Fe (2674 ppm), Mn (130 ppm) and Pb (334 ppm) in this grass species are in the vicinity of the closed Olkusz mine. These contents have increased in comparison with the 1994 figures: Fe by 56 %, Mn by 120 % and Pb by 6 %. In the birch leaves, the metal contents averaged for four sites are the following: As 2.1, Cd 6.5, Fe 261, Mn 110, Pb 70 and Zn 1657 ppm, being lower from the figures in 1994. The highest As, Fe and Pb contents of birch leaves occur in the habitat closest to the former Olkusz mine, while those of Cd, Mn and Zn in the habitat distant at 100 m from the active plant in Bukowno. The common bent grass generates better resistance mechanisms against the metals than does the birch. In the birch leaves, the contents of the metals are significantly lower than those of the grass blades, but higher from those of the birch seeds collected from the same tree individuals. It is a proof of good functioning of the mechanisms preventing excessive metal amounts from the cell metabolism and of the presence of physiological barriers protecting birch seeds as the generative organs.

## Introduction

Mining and metallurgy of Zn-Pb ores are examples of the industrial activities most devastating the environment (Verner et al. 1996; Kicińska 2011; Cabała 2009). Many years of geotechnical, hydrological and chemical transformations of the soil and plant cover, emissions of industrial dusts that are carriers of metals, for instance Zn, Pb, Cd, Tl and As, and accumulation of industrial waste disposed of on-mine dumps and in flotation-tailing ponds have led to permanent changes of biocenoses, particularly marked in the vegetation growing in the areas located close to Zn-Pb mines and processing plants (Landberg and Greger 1996; Kicińska-Świderska 2003; Larcher 2003). Clear effects of these changes have been recorded as inherited mechanisms of elevated tolerance to stress factors, which can be observed in common plant species (for instance, *Betula pendula*—further referred to as *Bp*, *Salix* L., *Agrostis capillaris*—further referred to as *Ac*) growing in the areas of Zn-Pb industrial activities and because of that highly polluted with heavy metals (Kayzer et al. 2011; Kicińska 2009; Szarek-Łukaszewska 2009). Such organisms, called metallophytes, are forced to adapt to environmental conditions they live in and become with time subject to microevolutionary changes. Thus, they show a significant diversification of their ontogenetic traits not only within a given taxon group but also within the same species (Nowak et al. 2011; Rivelli et al. 2014). Considering long-term impact of the metals accumulated in soils, the plants are expected to evolve into new forms and even taxa (Dueck et al. 1984; Ernst 2006; Wu et al. 1975).

A particular resistance and tolerance to high contents of metals in the environment are characteristic of grass (among others *Agrostis capillaris*, *Silene vulgaris*), moss (among others *Pleurozium schreberi*, *Sphagnum fallax*) and some pioneer tree species (among others *Betula pendula*, *Pinus silvestris*). The study carried out by Czerniak and Poszyler-Adamska (2006) shows that the assimilation systems of the *Bp* are predisposed to cumulate dioxins. Therefore, this species is often applied to monitor strongly polluted environments (Kayzer et al. 2011). The anatomical features of leaves, for example, their size, mass and the presence of necroses, may also be biomarkers of pollution with heavy metals and also of a water and/or nutrition deficit (Dmuchowski et al. 2011).

The amounts of elements accumulated in plants result from combined processes of their intake from soil solutions or a bedrock and absorption by aboveground plant parts. These processes are controlled, among others, by cation exchange through cellular membranes, intracellular ion transport, metabolism and finally the presence and interaction of particular ions in the rhizosphere (Kuo and Harsh 1997; Kabata-Pendias and Pendias 1999). However, essential in the studies on the impact of metals on plants in the areas highly contaminated by the Zn-Pb industry is not only analysing the total metal content of plants, but determining the bioavailable metal forms present in the environment and their ability to form complexes with some fractions of organic matter due to biosorption and cumulation processes (Chojnacka et al. 2005). A continuous monitoring of emissions polluting industrial areas allows measuring the annual deposition of contaminants in the sites more proximal or distant from ore mining and processing installations. However, the metals already accumulated in soils may still significantly affect living organisms for many years to come, despite limiting the environmental nuisance of current ore mining and processing (Astel et al. 2007).

An access to the European Union imposed on new country members introducing many legislative corrections, including the necessity of adapting industrial production to environmental requirements. It has brought about a significant, positive change of the volume and type of pollutants discharged into the environment. Therefore, the authors based on chemometric analyses to evaluate the changes of the chemical composition that took place during the last 20 years in the aboveground parts of two plants (*Bp* and *Ac*), commonly occurring in a close vicinity and in an areas more distant from the mining and metallurgical plants of the Olkusz ore area (OOA) of Poland. This study was focused on the following: (a) determining the current metal contents of selected plant species growing in the industrial areas affected by ages of intensive mining and metallurgical activity, (b) chemometric comparing the changes recorded over the last 20 years in the habitats of two common plant species—the *Ac* and the *Bp* and (c) identifying major contemporary sources of the metal-polluting soil-plant systems.

### Brief outline of Olkusz area of Zn-Pb ores

The OOA hosts one of the world-scale Zn-Pb deposits (*Mississippi Valley type*). It is located in the Silesia-Cracow industrial region of south-central Poland. The ore minerals of the OOA occur in ore-bearing dolostones in two forms: as primary sulphides ZnS—sphalerite, PbS—galena, FeS_2_—pyrite and marcasite, and secondary ‘oxides’ (carbonates, silicates, etc.) called also galmei ores (Sass-Gustkiewicz 1997). Mining of the Pb and Zn ores has proceeded in the region since the twelfth century; therefore, we face today the historical and contemporary environmental impacts of ore mining and processing. The area contains surface ore exposures and also disposal sites of flotation tailings, old dumps of slags and also active mining and metallurgical works ‘Bolesław’ further referred to as ZGH that produce, among others, lead products, electrolytic zinc and galvanization alloys. The whole OOA is exposed to a strong impact of that industry that affects its soil-plant system, surface waters, groundwaters and atmosphere. Emissions of SO_2_ and PM10 usually exceed standard values (DPE 2008/50/EC; DPE 2004/107/EC) and are really nuisances in the autumn and winter months (Table 1). Monitoring of the particulate matter that has been carried out since 2007 indicates its long-term and above-standard emissions, particularly of the PM10 fraction, but despite that, monitoring of the deposition of metals was interrupted some 15 years ago. The results of such measurements conducted in the 1990s by SANEPID (an independent domestic sanitary and epidemiological research and control institution) showed the following maximum values in the OOA (in mg/m^2^): Zn 2822, Cd 22, Pb 309 and Mn 62, also 7.2 g/m^2^ Fe_2_O_3_ (Table 2). This high annual load of metals falling onto the OOA soils must have exerted a considerable impact on all the organisms living within the area. A quality of habitats is particularly important for plants; the organisms that cannot change their locations. A growth of plants strongly depends on the living conditions, mainly stress factors (climate, macro- and microelements in the system, competitiveness of other organisms, etc.).

**Table 1:** Imissions of SO_2_ and PM10 recorded in the automatic air quality measuring station in Olkusz in years 2007–2014 (due to monitoring.krakow.pios.gov.pl)

Month	2007		2008		2009		2010		2011		2012		2013		2014	
	SO_2_	PM10	SO_2_	PM10	SO_2_	PM10	SO_2_	PM10	SO_2_	PM10	SO_2_	PM10	SO_2_	PM10	SO_2_	PM10
	(μg/m^3^)															
January	**28**	36	**30**	–	**40**	**69**	**36**	**67**	**24**	**79**	13	**45**	**37**	–	–	**44**
February	**30**	**57**	**26**	–	–	**53**	**36**	**71**	19	**71**	**37**	**107**	**27**	–	–	**58**
March	18	**60**	18	38	19	**42**	**23**	**49**	20	**77**	–	**53**	19	31	13	**42**
April	16	**52**	11	**44**	7	**41**	10	39	9	**46**	7	26	10	27	7	27
May	10	32	7	29	5	25	4	25	5	30	3	19	4	20	4	18
June	–	–	–	28	5	21	3	25	4	25	4	16	4	23	4	18
July	–	–	–	25	–	–	3	31	4	23	4	18	5	23	4	16
August	8	33	6	30	3	30	4	22	5	27	6	20	5	21	4	16
September	10	35	7	33	6	40	5	26	6	37	4	20	8	20	5	27
October	15	**51**	13	**50**	12	38	15	**42**	9	**48**	8	27	11	28	11	36
November	**32**	**55**	18	**44**	17	**47**	16	**44**	15	**83**	17	**44**	17	35	10	36
December	**41**	**70**	**23**	**52**	20	**55**	**49**	**94**	13	**54**	**35**	**62**	**24**	**44**	17	**41**
Annual average	20.8	48	14.3	37	14.8	42	17	45	11.1	50	12.5	38	14.3	27	10.3	32
Min.–max.	8–41	32–70	6–30	25–52	3–40	21–69	3–49	22–94	4–24	23–83	3–37	16–107	4–37	20–44	4–23	16–58

Bold values correspond to exceeding the allowable level of SO_2_: 20 μg m^−3^ and PM10: 40 μg m^−3^ [http://eur-lex.europa.eu/legal-content/PL/TXT/?uri=CELEX%3A32008L0050]

**Table 2:** Annual precipitation of dust and selected metals in Bukowno in 1995–1996 (*data from SANEPID station located in Bukowno*)

Annual precipitation	1995	1996
Dust (g/m^2^)	60	67
Zn (mg/m^2^)	1711	2822
Cd (mg/m^2^)	22.04	3.79
Pb (mg/m^2^)	203	309
Mn (mg/m^2^)	58	62
Fe_2_O_3_ (g/m^2^)	6.5	7.2

## Study site and methods

**Fig. 1:**
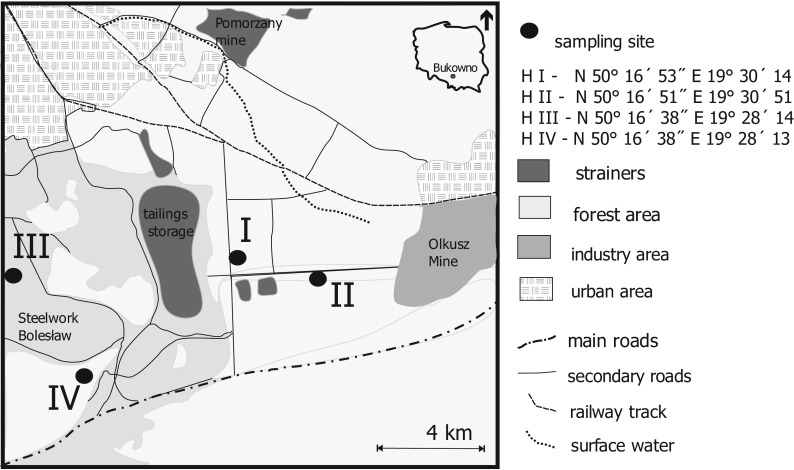
Sampling sites

The sites of the investigations carried out at the end of September 2014 were exactly the same as those of the analogue investigations that had been conducted in 1994 (Fig. 1, points I–IV); it allowed us to record long-term changes of the metal contents of some plants occupying these four sites. The habitats differ one from another first of all in their distance from and direction (wind rose) against major emitters (ZGH, flotation-tailing ponds and major transport routes). The research material collected in each of the sites included the following: 30 aboveground parts of the grass species *Ac* (symbols of samples G/I–G/IV), 1 kg of leaves of the tree species *Bp* (symbols BL/I–BL/IV) and 200–300 g seeds of the even-aged individuals of the pioneer tree *Bp* (symbols BS/I–BS/IV). The leaves of the birch trees from each habitat were divided into two parts. One of them was washed three times with 300 ml distilled water and next dried (symbol of samples BL_w_/I–BL_w_/IV); the other was not washed (symbol of samples BL_uw_/I–BL_uw_/IV).The contents of metals present on and inside the leaves were analysed. The load of metals (Cd, Fe, Mn, Pb and Zn) on leaves of the betula trees in 1994 and 2014 was calculated for the specified habitats as the difference between the metal contents of the unwashed and washed leaves (BL_UW_–BL_W_).

The total decomposition of plant tissue (both washed and not washed) was carried out with 7 ml of 65 % HNO_3_ and 1 ml of 30 % H_2_O_2_ in a microwave oven (high pressure-segmented rotor HPR 1000/10 s at 200 °C, microwave power up to 1000 W). Analytical weighed plant amounts were 0.5 g each. This methodology allows determining the total contents of metals in plants. The concentrations of metals (Zn, Pb, Cd, Fe and Mn) in solutions were measured with an ICP-MS 6100 analyser (Elan) at the detection limits of elements equal to 2°10^−5^ mg/dm^3^. Statistical analyses were carried out using the Statistica version 10 software (StatSoft Inc 2011).

## Results and discussion

### Metal contents of grass (common bent Ac)

The contents of As, Cd, Fe, Mn, Pb and Zn in samples of the common bent grass analysed in 2014 show significant differences among the four sites and low differences within a site (Table 3). The highest contents of Cd and Zn, 12 and 2524 ppm, respectively, were found in habitat no. III located in the closest distance about 100 m from ZGH. These figures compared with those obtained in 1994 are, however, lower by as much as 87 % for Cd and 52 % for Zn. The highest 2014 contents of Fe (2674 ppm), Mn (130 ppm) and Pb (334 ppm) have the grass samples from site no. II located in the closest distance from the Olkusz mine (open in 1962 and closed in 2003). Compared with those obtained in 1994, they are higher by 56, 120 and 6 %, respectively. In case of Zn and Pb, the 2014 figures for the *Ac* grass exceed significantly toxic limits (Table 3). The As content is the highest (17.8 ppm) in habitat no. I, which is 5 % higher than that analysed in 1994. In this site also higher are the 2014 contents of Mn (14 %) and Pb (12 %) in relation to the 1994 data. The lowest contents of the metals considered, ten times at the average if compared to three other sites, are those in the *Ac* growing in habitat no. IV located some 750 m from ZGH and to SW from the main pollution emitter.

**Table 3:** Concentration of metals in grasses (G) *Agrostis capillaris* in OOA

Kind of sample/no. of sampling site	As		Cd		Fe		Mn		Pb		Zn	
	1994^a^	2014	1994	2014	1994	2014	1994	2014	1994	2014	1994	2014
	(mg/kg)											
G/I	16.9	17.8 (+5)	10.5	2.9 (−72)	1303	1202 (−8)	37	42 (+14)	118	132 (+12)	844	414 (−51)
G/II	16.2	14.9 (−8)	27.5	5.5 (−80)	1716	2674 (+56)	59	130 (+120)	315	334 (+6)	2176	905 (−58)
G/III	26.6	7.1 (−73)	90.8	12.0 (−87)	3181	1161 (−64)	273	109 (−60)	1565	190 (−88)	5241	2524 (−52)
G/IV	4.8	0.8 (−83)	18.9	0.9 (−95)	1544	241 (−84)	138	31 (−78)	204	17 (−92)	1036	166 (−84)
AM	16.1	10.1 (−37)	36.9	5.3 (−86)	1936	1319 (−32)	127	78 (−39)	550	168 (−69)	2325	1002 (−54)
SD^c^	0.8		0.08		33		0.4		1.2		13	
CV^c^	0.08		0.01		0.03		0.01		0.01		0.01	
Natural content^b^	0.28–0.33		0.05–0.6		45–375		45–160		0.4–4.5		12–72	
Toxic content^b^	5		5–30		–		400–1000		30–300		100–400	

CV = SD/AM. The values in parentheses are calculated changes between years 1996 (100 %) and 2014 (data in %)

*AM* arithmetic mean, *SD* standard deviation (only for data from 2014), *CV* coefficient of variation

^a^Kicińska-Świderska (1999)

^b^Kabata-Pendias and Pendias (1999)

^c^Only for data from 2014

All the *Ac* samples have their As, Cd, Pb and Zn contents many times higher than the upper limits of the so-called natural grass contents (Kabata-Pendias and Pendias 1999). In the *Ac* from habitat nos. I, II and III are also exceeded the toxic limits of As and Zn that are 5 and 400 ppm, respectively. In the case of Pb with the toxic limit for grass 300 ppm, this value is exceeded (334 ppm, Pb) only in the *Ac* from the habitat no. II. In the *Ac* from habitat no. IV, the Fe content is below the upper natural value; while in the case of Mn, its values do not exceed such a value for all the four habitats. These results indicate significant adaptiveness and accumulation of metals in the species *Ac.*

In comparison with the 1994 data, the 2014 contents of metals in the aboveground *Ac* parts are significantly lower, with two exceptions only. The highest is the metal decrease in *Ac* (by 86 % on the average) in habitat no. IV, and the second in turn (a decrease of 70 % on the average) is habitat no. III. The lowest improvement (a decrease of only fifteen or so percent) has been found in habitat no. I. In the year 2014, the change of the habitat whose *Ac* had previously been most burdened with metals was also noted. It is habitat no. III, in which the significant improvement (70 % quoted above) is an effect of installing modern filters in the chimneys of the mining metallurical plant located nearby, due to which emission of industrial dusts has been considerably limited. The increases but in the cases of three metals and two habitats only in 2014 show the contents of Fe, Mn and Pb in habitats nos. I and II. This situation can be explained by a secondary deflation from large-size industrial dumps and old flotation tailing ponds. These waste materials still contain significant amounts of metals (Kicińska and Wójcik 2011; Gruszecka and Wdowin 2013), and the fine particles drift mainly to NE, i.e. in the prevailing direction of the wind rose, where habitat nos. I and II are located.

### Metal contents in birch trees Bp

The contents of metals in the leaves of the *Bp* analysed in 2014 are considerably lower than those obtained in 1994 (the same trees were sampled and at the same time of the year (Table 3); they are also lower from the metal contents of the grass *Ac* (Table 4).

**Table 4:** Concentration of metals in leaf birch washed (LB_W_) *Betula pendula* in OOA

Kind of sample/no. of sampling site	As		Cd		Fe		Mn		Pb		Zn	
	1994^a^	2014	1994	2014	1994	2014	1994	2014	1994	2014	1994	2014
	(mg/kg)											
LB_W_/I	12.3	0.1 (−99)	7.2	2.8 (−61)	744	210 (−72)	53	43 (−19)	77	52 (−32)	1478	613 (−59)
LB_W_/II	8.7	2.1 (−76)	18.2	2.0 (−89)	787	261 (−67)	148	105 (−29)	112	70 (−38)	3860	624 (−84)
LB_W_/III	9.5	1.7 (−82)	43.8	6.5 (−85)	1006	200 (−80)	145	110 (−24)	317	65 (−79)	2456	1657 (−33)
LB_W_/IV	9.6	b.l.d.	9.4	1.0 (−89)	303	159 (−48)	238	24 (−90)	69	11 (−84)	1486	478 (−68)
AM	10.2	0.7 (−93)	19.6	3.1 (−84)	710	207 (−71)	146	70 (−52)	144	49 (−66)	2320	843 (−64)
SD	±0.1		±0.00		±6.5		±2.8		±0.3		±45.0	
CV	0.13		0.00		0.03		0.04		0.01		0.05	

CV = SD/AM. The values in parentheses are calculated changes between years 1994 (100 %) and 2014 (data in %)

*b.l.d.* below level of detection, *AM* arithmetic mean, *SD* standard deviation, *CV* coefficient of variation

^a^Kicińska-Świderska (1999)

The highest 2014 metal contents of the birch *Bp* leaves are As 2.1, Cd 6.5, Fe 261, Mn 110, Pb 70 and Zn 1657 ppm. In the case of As, Fe and Pb, these values refer to habitat no. II, located in the closest distance from the Olkusz mine (closed in 2003), whereas in the case of Cd, Mn and Zn to habitat no. III, located only 100 m from ZGH. The lowest metal contents of the *Bp* leaves are in habitat no. IV; it is worth mentioning that also the lowest are metal contents of the *Ac* grass in the same habitat. Dmuchowski et al. (2011) analysed in 2010 the leaves of birch trees growing in the OOA and determined 0.11–9.95 ppm As (with an average for the whole OOA of 0.37 ppm). Their data correspond well with the figures presented here for the year 2014 (Table 4). The determinations of selected metals were also carried out in the birch leaves in 2005 (Gruszecka 2007); she found in the area of waste ponds the maximum contents of As 25, Cd 9.3, Cr 3.1, Pb 460 and Zn 1569 ppm. In both papers, the highest contents of As are noted in the birch leaves collected between ZGH and the former Olkusz mine.

A significant decrease of the As, Cd, Fe, Mn, Pb and Zn contents of the *Bp* leaves is the most important conclusion resulting from the comparison of the 1994 and 2014 data. The highest improvement is in the case of As, whose averaged content for all the habitats dropped by 93 %. For other metals, such drops are the following: Cd by 84 %, Fe by 71 %, Pb by 66 %, Zn by 64 % and Mn by 52 %. It is a desirable tendency of environmental changes noted in the 20-year period.

Seeds were the other research material collected from the birch trees. The highest contents of the metals in the seeds occur in habitat no. III (Table 5). They are as follows (the italicised values in parentheses show corresponding contents of leaves): Cd 5.4 ppm (*6.5*), Mn 96 ppm (*110*), Pb 46 ppm (*65*) and Zn 848 ppm (*1657*). Although these seed contents of Cd, Fe, Pb and Zn are lower (even by around 90 %) in comparison to the 1994 figures, habitat no. III has been still the most contaminated of the four sites tested. In contrast, in the seeds of habitat no. II, the contents of Pb increased by 9 % and those of Mn even by 91 %, referred to the 1994 figures, but the remaining elements noted significant drops: Cd by 91 %, Fe by 46 % and Zn by 57 %. The lowest metal contents of the birch tree seeds are in habitat no. IV.

**Table 5:** Concentration of metals in birch seeds (BS) *Betula pendula* in OOA

Kind of sample/no. of sampling site	Cd		Fe		Mn		Pb		Zn	
	1994^a^	2014	1994	2014	1994	2014	1994	2014	1994	2014
	(mg/kg)									
BS/I	5.8	2.1 (−64)	551	185 (−66)	64	23 (−64)	33	16 (−51)	604	182 (−70)
BS/II	22.1	1.9 (−91)	351	191 (−46)	46	88 (+91)	22	24 (+9)	555	238 (−57)
BS/III	346.0	5.4 (−98)	2770	346 (−87)	207	96 (−54)	2481	46 (−98)	9193	848 (−91)
BS/IV	3.5	1.7 (−50)	632	139 (−78)	57	28 (−51)	33	7 (−79)	200	231 (+15)
AM	94.3	2.8 (−97)	1076	215 (−80)	93	59 (−37)	2544	23 (−99)	2638	375 (−86)
SD	±0.04		±0.4		±0.5		±0.4		±3.2	
CV	0.01		0.00		0.01		0.02		0.01	

CV = SD/AM. The values in parentheses are calculated changes between years 1994 (100 %) and 2014 (data in %)

*AM* arithmetic mean, *SD* standard deviation, *CV* coefficient of variation;

^a^Kicińska-Świderska (1999)

Considering the metal contents of the birch seeds averaged for all the habitats, the 1994:2014 decreases are high: Pb by 99 %, Cd by 97 %, Zn by 86 %, Fe by 80 % and Mn by 37 %.

The contents of the metals in the birch seeds are two to three times on the average lower if compared with the metal contents of the leaves collected from the same trees (Table 4). It is a proof of a predominance of a passive (non-metabolic) transport of elements, particularly of Zn and Pb, over the active transport, based mainly on the diffusion of Pb^2+^ and Zn^2+^ ions from a soil solution into the endodermic cells of the plant roots, from which they are transported into the aboveground plant parts by a transpiratory water movement. A differentiation of the contents of elements in the generative and vegetative plant parts results from a metabolic (active) uptake of metals by root systems. The differences may be due to either the lack of a plant biologic barrier, which leads to a fast adaptation of such a species, or the inherited resistance strategy of a plant organism based on retaining metals in the roots. There are practically two mechanisms of detoxication acting in root systems. In the first of them, the metals are accumulated in the cell walls; in the other, they are bound by phenol compounds in the vacuoles of tannin cells. Such mechanisms were also found in birch trees by Ciarkowska et al. (2009), who determined that the roots of the birches growing on the substrate highly polluted with flotation tailings from the OOA took up 8 % Zn, 4 % Pb and 1 % Cd of the metals present in the substrate, whereas the analogue uptake of these metals by birch sprouts was lower by about 1 %. When the birch trees grew on a non-polluted substrate, the roots retained 75 % Zn, 24 % Pb and 72 % Cd% present in their substrate, and the respective uptakes of the three metals in the aboveground birch parts was significantly lower.

In the case of the *Bp*, high amounts of metals taken up from soil solutions are stopped by both the tree root systems and the physiological barriers due to which the metals are immobilised in the vegetative plant organs, while the generative organs, i.e. seeds, are protected. This process has been traced using the 1994 and 2014 data by calculating the transport index *T*: it is a quotient of the content of a given element in the birch leaves (vegetative parts) to such content in the birch seeds (generative parts). The values of the index *T* ≤ 1 indicate that the physiological barriers in the aboveground birch parts do not retain excessive amounts of elements. If *T* > 1, the retaining mechanisms are active in the roots. The results of such calculations (Table 6) show that in 1994 the worst situation was in habitat no. III, for which the *T* indices took on the values significantly below 1. It is particularly worrying in the case of Cd and Pb with the *T* = 0.13. Such a low index indicates a weak action of the mechanisms protecting plants from excessive amounts of toxic elements. The highest *T* indices have been calculated for habitat no. IV in the case of Cd and Zn.

**Table 6:** Coefficient *T* for sampling sites in 1994 and 2014

Sampling site	Cd	Fe	Mn	Pb	Zn
*T*—1994					
I	1.24	1.35	0.83	2.33	2.45
II	0.82	2.24	3.22	5.09	6.95
III	0.13	0.36	0.70	0.13	0.27
IV	2.69	0.48	4.18	2.09	7.43
Average	1.22	1.11	2.23	2.41	4.27
*T*—2014					
I	1.31	1.14	1.87	3.27	3.37
II	1.07	1.37	1.18	2.97	2.62
III	1.19	0.58	1.14	1.40	1.95
IV	0.58	1.15	0.84	1.57	2.07
Average	1.04	1.06	1.26	2.30	2.50

*T* quotient of the metal content in birch leaf in relation to the content in birch seeds

Considering the 2014 data, the *T* index is improved for all the metals, with the highest change of its values even by 1.77 units. Such results point to developing and fixing by the birch trees protecting mechanisms adequate to the existing environmental conditions.

### Current sources of metals affecting the OOA plant species

**Fig. 2:**
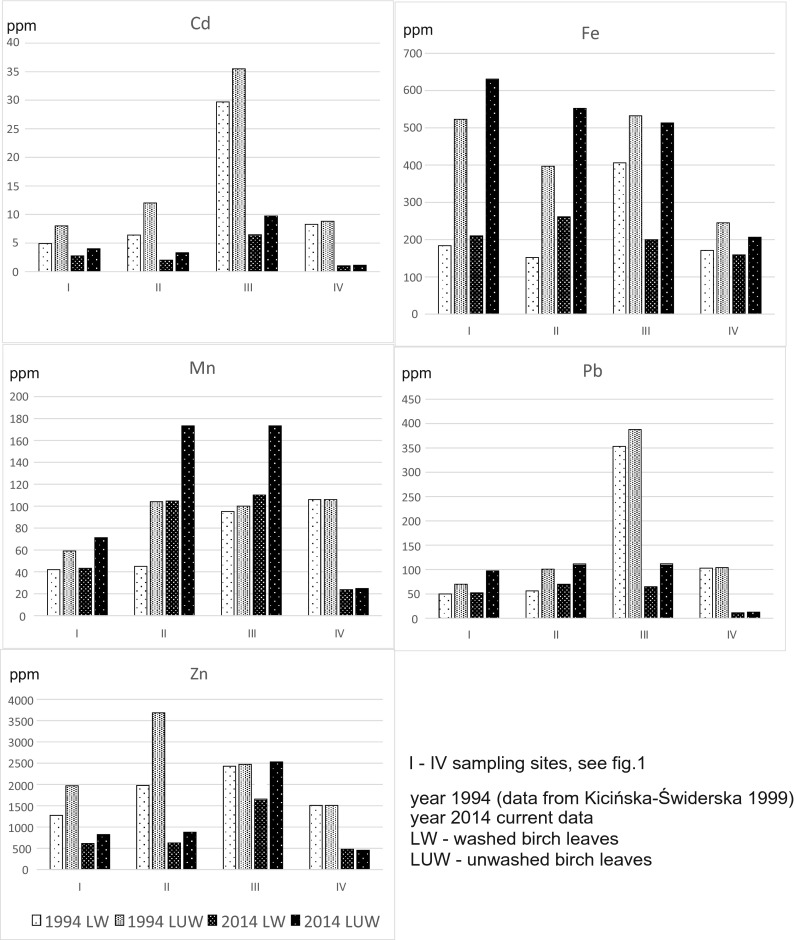
Metal content in betula leaves (washed and unwashed) in 1994 and 2014

Up to now, two sources of metals affecting the plants growing in the OOA have been indicated. The first is the so-called soil load, understood not only as the total content of metals, but as the bioavailable forms of ions present in the soil environment. High amounts of metals in the soil affect the morphology of roots: they dry, get deformed, have the growth limited and are deprived of symbiotic mycelia. These are results of a disturbed histogenesis, improper development of lateral roots or unbalanced tissue growth (Cabała 2009). The other source is represented by the so-called atmospheric load, understood as the total of various substances: polar (gases and dust) and nonpolar (mainly organic compounds) polluting the air (Poborski 1991; Kicińska-Świderska 2004). If deposited on the surfaces of the aboveground plant fragments, they can enter their inner parts via a stomatal and/or extrastomatal uptake and be included in (or partly excluded from) plant metabolic processes. Mechanisms of a fast excluding the excessive, free ions that enter plant cells via a metabolic route result in the formation of its tolerance to some elements. Such a process depends on a plant species, its ontogenic traits and the type of the element (Ernst 2006). The amounts of metals that can be present on aboveground plant parts were calculated comparing the metal contents in washed and not washed samples of birch leaves. The results for the years 1994 and 2014 (Fig. 2) indicate the following:

- a few times (from 2 to 5) decrease of the Cd and Zn amounts present on birch leaves between 1994 and 2014,
- habitat no. III, in which the highest decrease of the contents Pb and Cd both in washed and not washed leaves was recorded between 1994 and 2014 and
- habitat nos. I and II, in which the contents of Fe and Mn in the not washed leaves increased in 2014 in relation to those in 1994.

**Fig. 3:**
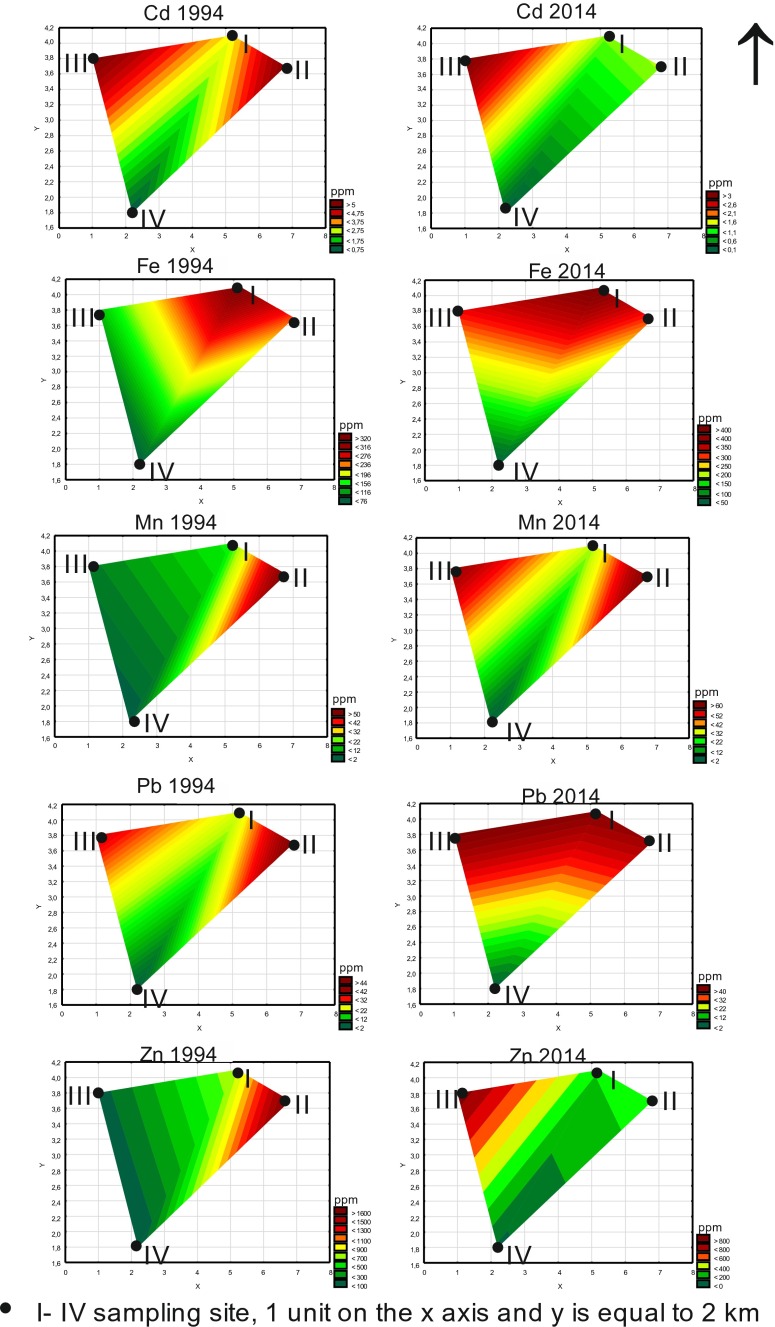
Load of pollutants on the betula leaves of the considered OOA habitats in 1994 and 2014

These results point to a long-lasting and intensive deflation resulting in drifting the dusts containing high contents of metal compounds (Fe, Mn) from the flotation tailing dried ponds located in proximity to the habitats tested (Kicińska-Świderska 1999; Gruszecka and Wdowin 2013). The samples from the closest vicinity of the ZGH (habitat nos. III and IV, Fig. 1) indicate a significant environmental improvement, i.e. a decrease of the amounts of the deflated dusts containing metals and deposited onto soils. It confirms positive effects of the actions limiting the environmental impact of the mining and processing ZGH installations. To obtain a better visualisation of the inflow direction of pollutants via the atmosphere, the Wafer 3 W (XYZ) plots have been prepared, ascribing the grid location coordinates of the habitats adequate rectangular coordinates. The load of metals on leaves of the betula trees in 1994 and 2014 was calculated for the specified habitats as the difference between the metal contents of the unwashed and washed leaves (Fig. 3). The graphs show the direction(s) of the maximum inflow of the pollutants deposited on plants.

In the year 1994, the highest loads of atmospheric pollutants affected the plants growing in the eastern part of the OOA. The most polluted with Zn, Pb, Mn, Fe and Cd were the aboveground parts of the plants of habitat no. II, located in the closest distance from the active then Olkusz mine. In the case of Pb and Cd, their additional source was represented by secondary dusts transported by the western winds prevailing in the OOA region. It can be thus assumed that these pollutants originated due to operations of the ZGH. In the year 2014, an important change was recorded, i.e. a decrease of the Zn and Cd amounts deposited on aboveground parts of the plant. The plants of habitat no. III were exposed most on the deposition of these two metals. The remaining metals were transported by winds from the north, which is a new direction, in which old ponds of flotation tailings are located and are currently a significant source of metals that pollute also plants nearby. In the case of Fe and Mn analysed in 2014, their contents are higher than those recorded in the 1990s. The least polluted in 1994 was habitat no. IV, which has also now been the cleanest of the four habitats. Although located 700 m from ZGH, this site is situated south of the Zn-Pb works that is upwind from a major pollution source.

## Conclusions

The metal contents in the common bent *Ac* were significantly higher in 2014 than the values published as the so-called natural contents. In all habitats, these values also exceed the contents regarded as toxic ones. It is a proof that the *Ac* grass has developed permanent tolerance particularly to high contents of Zn, Pb and Cd. Despite considerable metal amounts, the *Ac* grass individuals have not revealed visible changes of their aboveground morphology, and the in situ inspections have shown their normal growth.

The *Ac* grass individuals also contain higher metal contents in comparison to those of the pioneer *Bp* birch tree, which suggests that the resistance mechanisms against pollution of grass varieties exceed those of trees. The metal contents of the birch are higher than those of the birch seeds collected from the same trees. It is, in turn, a sign of a proper functioning of the mechanisms that prevent excessive metal amounts from entering into the cell metabolic system and of the presence of physiological barriers protecting the generative organs of trees. A comparison of the 1994 and 2014 results shows that a long-lasting impact of stress factors on plants results in their developing and fixing some protection mechanisms that become genetically transferred to next generations. The fact that the active metal transport is dominating in the case of generative parts of plants indicates that concentrations of ions in soil solutions and amounts of elements in atmospheric dusts significantly decreased between 1994 and 2014.

Preventive measures and legal regulations pertaining to the environmental protection and introduced in the last 20 years have considerably improved a state of the environment in the Olkusz ore area (OOA). The changes are clearly marked in vicinity of the ZGH plant, whose emissions have been limited. Of the four habitats studied, the soil-plant structure of the habitat located in the shortest distance from ZGH has changed the most and its pollution distinctly dropped. However, the study has identified new sources of metal emissions: they come from unprotected (or insufficiently protected) old ponds of flotation tailings and slag dumps. These are the sites currently contaminating the regions located to the south and southeast of them, i.e. in the direction of dominant winds that deflate fine, metal-containing dust particles.
